# Application of *Bacillus* spp. Phosphate-Solubilizing Bacteria Improves Common Bean Production Compared to Conventional Fertilization

**DOI:** 10.3390/plants12223827

**Published:** 2023-11-11

**Authors:** Antonia Erica Santos de Souza, Vinicius Augusto Filla, João Paulo Morais da Silva, Marcelo Rodrigues Barbosa Júnior, Christiane Abreu de Oliveira-Paiva, Anderson Prates Coelho, Leandro Borges Lemos

**Affiliations:** 1 School of Agricultural and Veterinary Sciences, São Paulo State University (UNESP), Jaboticabal 14884-900, SP, Brazil; antonia.erica@unesp.br (A.E.S.d.S.); vinicius.filla@unesp.br (V.A.F.); joao.morais-silva@unesp.br (J.P.M.d.S.); marcelo.junior@unesp.br (M.R.B.J.); leandro.lemos@unesp.br (L.B.L.); 2 Embrapa Corn and Sorghum, Sete Lagoas 35701-970, MG, Brazil; christiane.paiva@embrapa.br

**Keywords:** *Bacillus subtilis*, *Bacillus megaterium*, *Phaseolus vulgaris* L., phosphorus, sustainability

## Abstract

The use of phosphate-solubilizing bacteria (PSB) can be a sustainable strategy to increase phosphorus availability and promote satisfactory crop yields. The objective of this study was to evaluate whether inoculation with PSB in common bean increases (i) growth, (ii) nutrition, (iii) yield, and (iv) grain quality, and (v) reduces the chemical phosphorus application dose to obtain maximum yields. The experiment was conducted in an Oxisol using a randomized block design in a 4 × 4 factorial scheme, with four replicates, using the cultivar IAC 2051. The first factor was four doses of P_2_O_5_ (0, 20, 40 and 60 kg ha^−1^), and the second factor was four doses of PSB (0, 100, 200 and 300 mL ha^−1^). For leaf area and leaf chlorophyll content, the association of PSB inoculation with a P_2_O_5_ dose of 40 kg ha^−1^ promoted the best conditions for the common bean. P_2_O_5_ application increased yield by 79 kg ha^−1^ for each 10 kg ha^−1^ added. PSB inoculation at a dose of 192 mL ha^−1^ promoted P export of 15.3 kg ha^−1^, and the PSB dose of 159 mL ha^−1^ increased yield by 389 kg ha^−1^ (12%) compared to the control. Grain quality remained within the standards required by the consumer market, being little affected by the treatments. Improvements in common bean growth and nutritional and physiological status promoted by P_2_O_5_ application and PSB were essential in increasing yield, so these are sustainable production strategies.

## 1. Introduction

The common bean (*Phaseolus vulgaris* L.) is an annual cycle legume that produces edible grains of high nutritional value [1]. Approximately 20% of the dry weight of grains is protein, and they are also rich in vitamins, carbohydrates and minerals [2]. This crop can be used to compose different agricultural production systems due to its benefits within the supply of soil fertility, improvements in soil quality and short cycle [3].

Nitrogen (N) and phosphorus (P) are the most limiting nutrients that sustain grain yield. N acquisition by common beans is favored by symbiotic associations with Rhizobium spp., capable of fixing atmospheric N [4]. This association contributes to N management and a reduction in the use synthetic N fertilizers. There are several studies regarding sustainable and more economical strategies for supplying N to common beans [5,6,7]; a combination of superior strains and minimal N fertilizer application has shown significant increase in common bean yield [8,9]. But few studies address the efficiency of P in this crop. Thus, more sustainable strategies for P supply to common beans should be analyzed [2].

Phosphorus is one of the most limiting nutrients for crop yield. In tropical regions, due to the process of P immobilization via soil clay fraction, this is even more pronounced [10]. In these regions, most soils have a high degree of weathering and they lack this nutrient due to the processes of adsorption to the clay fraction of the soil, which make this nutrient unavailable for absorption by plants [10]. Brazilian agricultural soils are known for their high capacity to fix P due to their pedogenesis, characterized by high concentrations of Al and Fe oxides, which favor the processes of unavailability of this nutrient [11].

The lack of P in soils compromises processes such as photosynthesis and respiration of plants, which respond morphologically, with low growth and yield [12], in addition to compromising biological N fixation (BNF) [12]. To overcome the limited availability of P in soils, high doses of phosphate fertilizers are imported and applied to sustain crop yield, putting Brazil in a position of risk due to high dependence on the import of chemical fertilizers [13]. Geopolitical factors can generate insecurity in the acquisition of this input and increase production costs [13]. Therefore, new alternatives are needed to satisfy the acquisition of P by plants, aiming to improve agricultural systems.

Microorganisms have great importance within biogeochemical cycles, particularly that of P, so they have been pointed out as an independent or supplementary technology to phosphate chemical fertilizers [14]. The mechanisms of bacterial activity in the P cycle have been well documented, and these species are termed phosphate-solubilizing bacteria [15,16]. The genus *Bacillus* spp. is one of the most dominant in this effect. These bacteria act on the forms of P in the soil, solubilizing the inorganic portion through the release of organic acids or through the mineralization of the organic fraction by the exudation of enzymes [17]. Katsenios et al. [18] observed that *Bacillus subtilis* application to sweet corn increased its productivity by 13.8% compared to the control. Evaluating soybean yield depending on the application of *Bacillus subtilis* and *Bacillus megaterium*, Leite et al. [19] observed increases of 813 kg ha^−1^ compared to the control with standard inoculation alone.

Recent studies have found that PSB application improves the growth, nutrition and yield of cereals, such as maize [20], wheat [21] and rice [10], as well as legumes, such as soybeans [22]. Plants have different P requirements during their growing cycle. An integrated study of PSB and phosphate fertilizer provides information on whether bacteria are able to meet the total P requirement of plants or partially depend on phosphate fertilizers [23]. In addition, inoculation rates may also have an influence on the efficiency of bacteria in the plant–soil system [24]. Therefore, efforts should be made to determine the appropriate rates of PSB and phosphate fertilization in the field in order to find the appropriate proportions for correct P nutrition in common beans. Efficient and sustainable management of P poses a challenge to achieving the second sustainable development goal [25].

Thus, this study aimed to evaluate whether inoculation with phosphate-solubilizing bacteria in common beans increases (i) growth, (ii) nutrition, (iii) yield and (iv) grain quality and, (v) reduces the chemical phosphorus application dose to obtain maximum yields.

## 2. Results

### 2.1. Phosphorus Content and Accumulation in Common Beans

P_2_O_5_ doses and PSB inoculation altered P content and accumulation in common beans (Supplementary Table S1). P_2_O_5_ application and phosphate-solubilizing bacteria (PSB) inoculation effectively contributed to the increase in P content in the dry matter (PCDM), whereas for the variables P contents in leaves (PCL) and P contents in grains (PCG), only the application of P_2_O_5_ was efficient. In contrast, significant responses to PSB inoculation were achieved for P accumulation in the dry matter (PADM) and P export. The study factors did not affect the agronomic efficiency of common beans.

**Figure 1:**
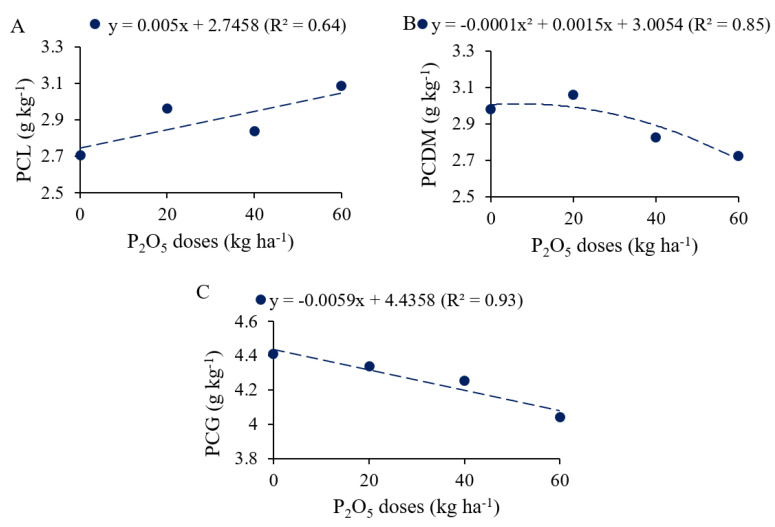
P content in leaves (PCL, (**A**)), dry matter (PCDM, (**B**)) and grains (PCG, (**C**)) of common bean as a function of P_2_O_5_ doses.

PCL increased linearly at a rate of 0.05 g kg^−1^ for each 10 kg ha^−1^ of P_2_O_5_ added (Figure 1A). PCDM showed quadratic variation as a function of the increase in P_2_O_5_ doses, with a maximum value of 2.96 g kg^−1^ when plants were fertilized with 5.6 kg ha^−1^ of P_2_O_5_. However, PCG decreased linearly at a rate of 0.059 g kg^−1^ for each increase of 10 kg ha^−1^ of P_2_O_5_.

**Figure 2:**
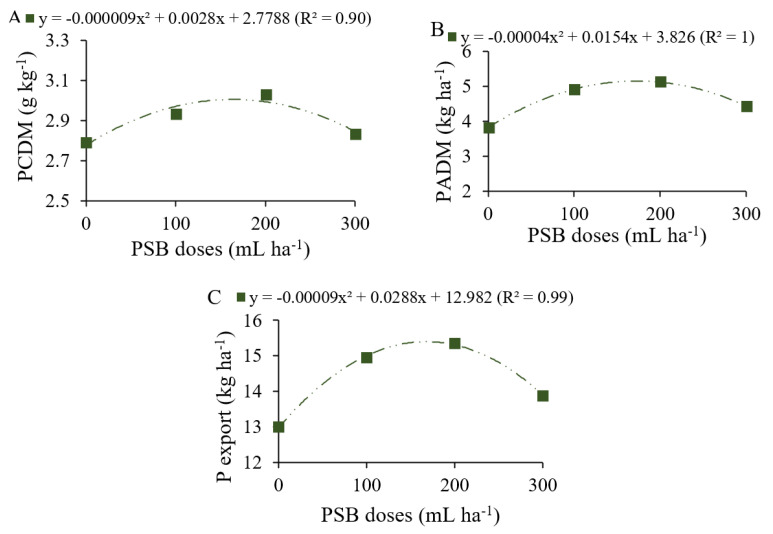
P content in the dry matter (PCDM, (**A**)), P accumulation in the dry matter (PADM, (**B**)) and P export (**C**) by common beans as a function of phosphate-solubilizing bacteria (PSB).

PCDM showed quadratic variation as a function of PSB doses, with a maximum content of 2.99 g kg^−1^ at a PSB dose of 156 mL ha^−1^ (Figure 2A). In addition, PADM and P export also showed quadratic variation as a function of PSB doses (Figure 2B,C). The maximum values for PADM (5.30 kg ha^−1^) and P export (15.28 kg ha^−1^) were found at PSB doses of 160 and 192 mL ha^−1^, respectively.

### 2.2. Chlorophyll Content Index and Photosynthetic Pigments

There was an interaction between the P_2_O_5_ doses and PSB doses for the photosynthetic pigments Chl a, Chl b and Cx + c in the vegetative stage (V4). The effect of the P_2_O_5_ doses was significant for Chl total (Supplementary Table S2). In the reproductive stages the effect of the P_2_O_5_ doses was significant for the chlorophyll content index (CCI) in R6 and R8, whereas for the pigments Chl a, Chl b and Chl total differences were observed only in R8 (Supplementary Table S2).

**Figure 3:**
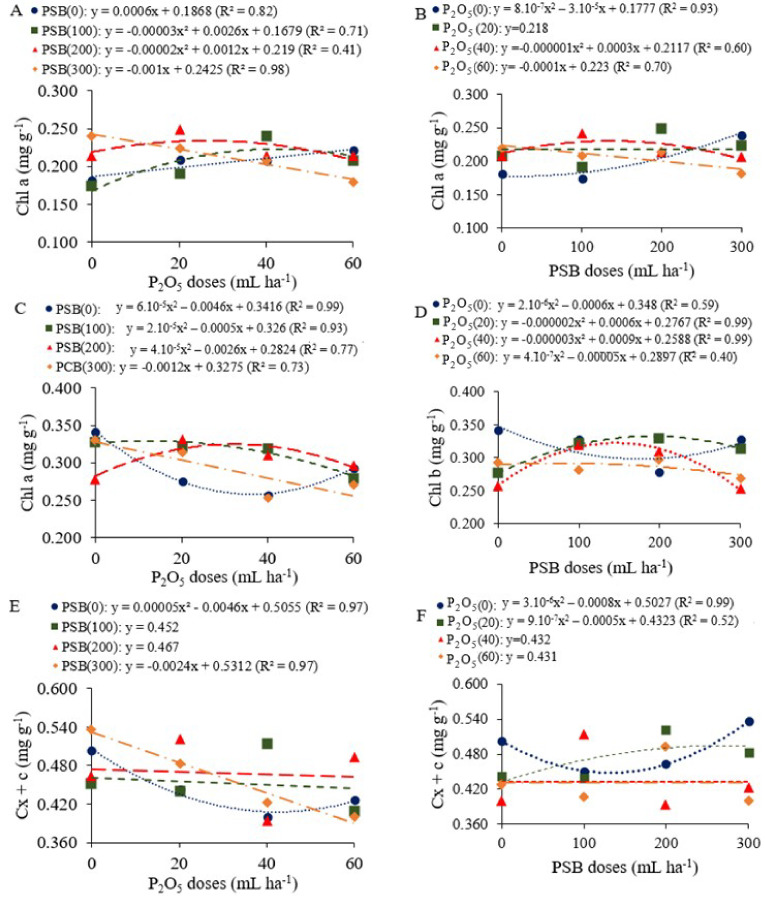
Decomposition of the interaction between P_2_O_5_ doses and phosphate solubilizing bacteria (PSB) doses for chlorophyll a (Chl a, (**A**,**B**)), chlorophyll b (Chl b, (**C**,**D**)) and carotenoids (Cx + c, (**E**,**F**)) in common bean plants in the vegetative stage (V4).

The content of chlorophylls and carotenoids responded significantly to PSB doses combined with P_2_O_5_ in the stage V4 (Figure 3). Chl a production, as a function of phosphate fertilization, increased linearly when common bean plants were not inoculated. When inoculated with doses of 100 and 200 mL ha^−1^, plants showed a quadratic response, with maximum values of 0.224 and 0.237 mg g^−1^ after fertilization with P_2_O_5_ doses of 43.3 and 30.0 kg ha^−1^, respectively (Figure 3A). As a function of the PSB doses, Chl a decreased quadratically when common bean plants were not fertilized. For this treatment, higher amounts of this pigment were obtained with PSB doses above 18.75 mL ha^−1^. After fertilization with doses higher than the recommended (40 kg ha^−1^ of P_2_O_5_), this group of pigments decreased quadratically when plants were inoculated with PSB doses greater than 150 mL ha^−1^ (Figure 3B).

As a function of phosphate fertilization, when common bean plants were not inoculated, Chl b production decreased quadratically up to the P_2_O_5_ dose of 38.3 kg ha^−1^. When plants were inoculated with 100 and 200 mL ha^−1^ of PSB, the highest Chl b contents were observed at P_2_O_5_ doses of 12.5 and 32.5 kg ha^−1^, respectively (Figure 3C). As a function of inoculation, after fertilization with doses lower than the recommended (40 kg ha^−1^ of P_2_O_5_), the maximum production of this pigment was reached when common bean plants were inoculated with up to 150 mL ha^−1^ of PSB (Figure 3D). When not inoculated, it decreased after fertilization with P_2_O_5_ up to a dose of 46 kg ha^−1^, and at a dose of 300 mL ha^−1^ this group of pigments decreased linearly (Figure 3E).

As a function of the PSB doses, the contents of carotenoids decreased up to a dose of 133.3 mL ha^−1^ when common bean plants received no phosphate fertilization. Under reduced fertilization (20 kg ha^−1^ of P_2_O_5_), this group of pigments showed a quadratic variation, with the maximum value obtained after inoculation with up to 278 mL ha^−1^ of PSB (Figure 3F).

**Figure 4:**
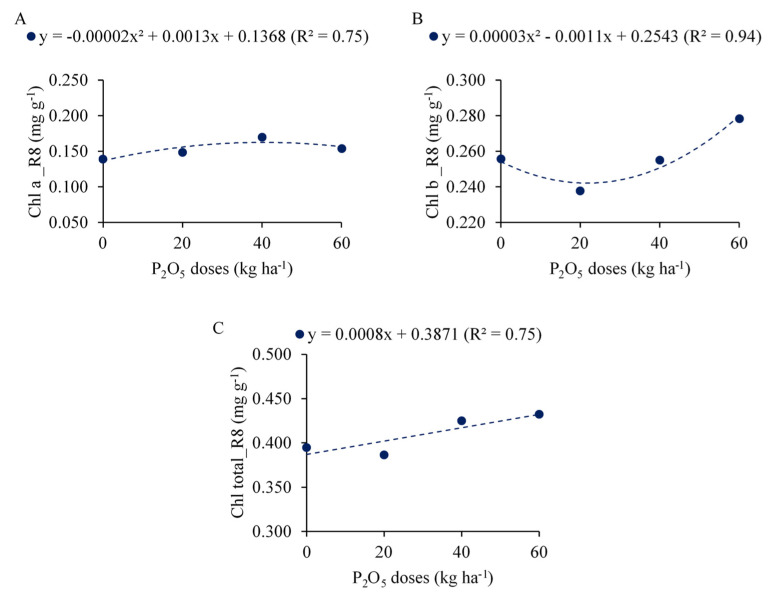
Chlorophyll a (Chl a, (**A**)), chlorophyll b (Chl b, (**B**)) and chlorophyll total (Chl total, (**C**)) in common bean plants in stage R8 as a function of P_2_O_5_ doses.

As a function of P_2_O_5_ doses, Chl a increased quadratically, with a maximum concentration of 0.157 mg g^−1^ at a dose of 32.5 kg ha^−1^ (Figure 4A). Chl b decreased quadratically up to 0.244 mg g^−1^ at the dose of 18.3 kg ha^−1^, respectively (Figure 4B). Regarding the total chlorophyll content, a linear increase in this group of photosynthetic pigments was observed as a function of the P_2_O_5_ doses at a rate of 0.008 mg g^−1^ for each 10 kg ha^−1^ added (Figure 4C).

**Figure 5:**
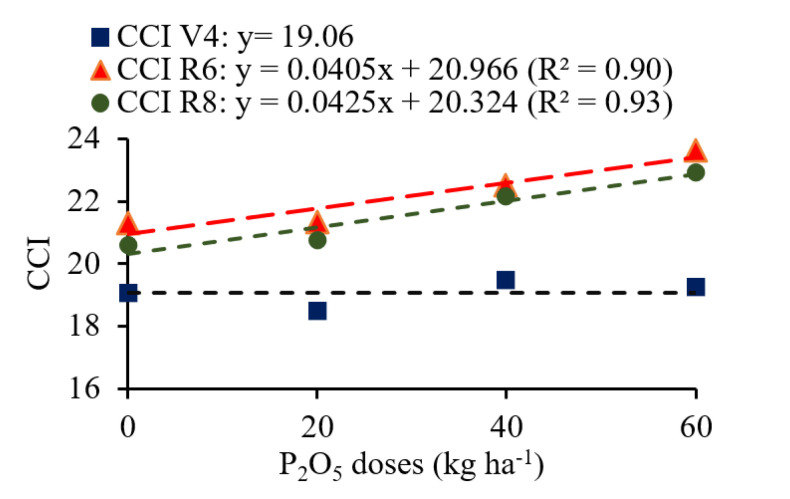
Chlorophyll content index (CCI) in common bean plants as a function of P_2_O_5_ doses.

Linear increments were observed for the CCI in stages R6 and R8 at rates of 0.405 and 0.425 for each 10 kg ha^−1^ of P_2_O_5_ added. In stage V4, the CCI was not influenced by the P_2_O_5_ doses, but lower values were observed compared to the reproductive stages. In general, at the R6 stage, the plants had higher CCIs compared to the others (Figure 5).

### 2.3. Growth, Production and Yield Components

Leaf area was influenced by the interaction between the P_2_O_5_ and PSB doses (Supplementary Table S3). For number of pods per plant (NPP) and number of grains per pod (NGP), there were influences of the inoculant doses as single factors, regardless of the phosphorus doses applied. Dry matter (DM) and yield (YLD) were affected by the main effects, i.e., P_2_O_5_ doses and inoculation with PSB doses (Supplementary Table S3). Hundred-grain weight (HGW) was not affected by P_2_O_5_ doses, inoculant doses or by the interaction between factors (Supplementary Table S3).

**Figure 6:**
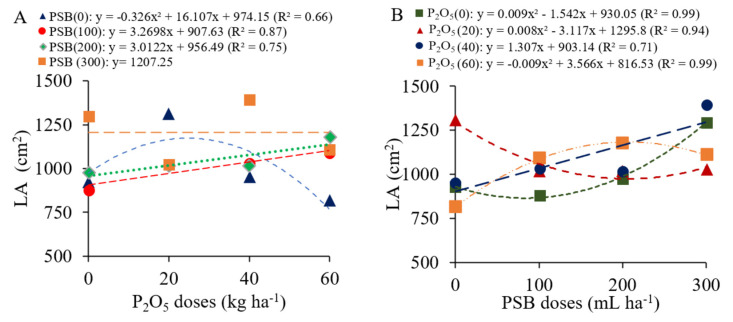
Interactions between P_2_O_5_ doses (**A**) and phosphate-solubilizing bacteria doses (PSB, (**B**)) for leaf area (LA) of common beans.

When common bean plants did not receive PSB, their leaf area increased quadratically in response to P_2_O_5_ doses. Under inoculation with 100 and 200 mL ha^−1^ it increased linearly, whereas under inoculation with 300 mL ha^−1^ this variable did not respond to the P_2_O_5_ doses (Figure 6A). Regarding the P_2_O_5_ doses as a function of the increasing doses of PSB, at the minimum dose of P_2_O_5_ the common bean plants needed PSB doses greater than 85.7 mL ha^−1^ to produce a larger leaf area, while the highest dose of P_2_O_5_ resulted in a larger leaf area when plants were inoculated with PSB doses of up to 198.1 mL ha^−1^ (Figure 6B).

**Figure 7:**
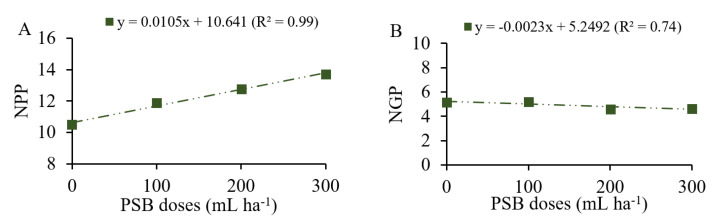
Number of pods per plant (NPP, (**A**)) and number of grains per pod (NGP, (**B**)) as a function of phosphate-solubilizing bacteria (PSB) doses.

NPP increased linearly at a rate of 1.05 for each 100 mL ha^−1^ of PSB added (Figure 7A). NGP decreased linearly with the increasing doses of inoculant at a rate of 0.23 for each 100 mL ha^−1^ of PSB added (Figure 7B). In summary, plants with fewer pods had a higher number of grains per pod produced as a function of the increasing doses of P_2_O_5_.

**Figure 8:**
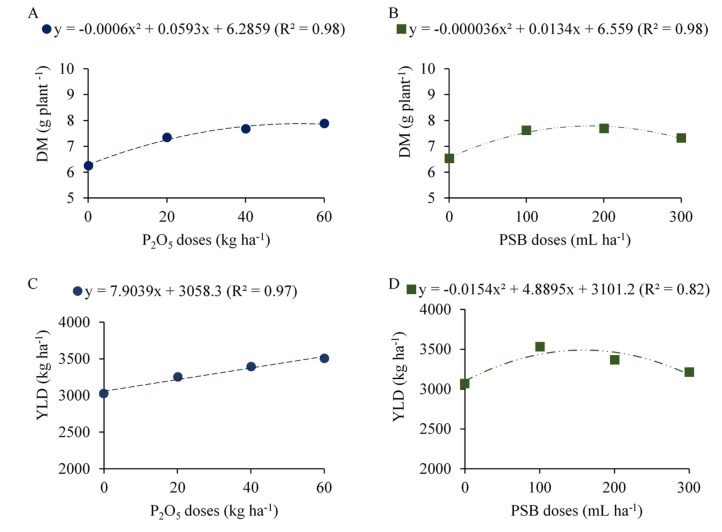
Dry matter (DM, (**A**,**B**)) and yield (YLD, (**C**,**D**)) of common bean plants as a function of P_2_O_5_ doses and phosphate-solubilizing bacteria (PSB) doses.

DM increased quadratically, with a maximum value of 7.75 g plant^−1^ with the addition of 49.4 kg ha^−1^ of P_2_O_5_ (Figure 8A). For the PSB doses, the increase was also quadratic, with a maximum value of 7.80 g plant^−1^ at the dose of 186.1 mL ha^−1^ (Figure 8B). YLD showed a linear increase at a rate of 79 kg ha^−1^ for each 10 kg ha^−1^ of P_2_O_5_ added (Figure 8C) and a quadratic variation for PSB doses. For the latter case, the maximum YLD of common bean was 3489 kg ha^−1^ when plants were inoculated with a PSB dose of 159 mL ha^−1^ (Figure 8D).

### 2.4. Common Bean Grain Quality

For the qualitative attributes of the common bean, only the cooking time (CKT) was influenced by P_2_O_5_ doses (Supplementary Table S4). When plants were fertilized with P_2_O_5_ up to the dose of 29.7 kg ha^−1^, the cooking time of the grains decreased quadratically up to 10.16 min. Under fertilization with doses higher than that, CKT tended to be higher (Supplementary Figure S3). The mean YS ≥ 12 of the experiment was 85.7%, with CPC of 17.0%, TMH of 15:25 (h:min) and HR of 2.02.

### 2.5. Multivariate Principal Component Analysis (PCA)

**Figure 9:**
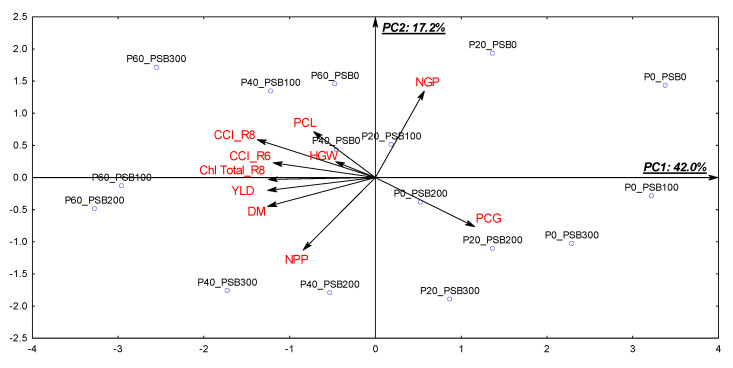
Principal component analysis for spatialization of treatments and variables in two dimensions (biplot). PC: principal component; P: P_2_O_5_ doses; PSB: phosphate-solubilizing bacteria; YLD: grain yield; NPP: number of pods per plant; NGP: number of grains per pod; HGW: hundred-grain weight; DM: aerial part dry matter; CCI: chlorophyll content index; Chl total: total chlorophyll content in the leaves; PCL: P content in the leaves; PCG: P content in the grains.

The first two principal components were responsible for explaining approximately 60% of the total variability of the data (Figure 9). The variables correlated with the first principal component (PC1) were the CCI in R6 (−0.74), the CCI in R8 (−0.84), Chl total in R8 (−0.76), DM (−0.78), YLD (−0.77) and PCG (0.71). Variables with equal signs of the factor loadings are directly correlated with each other and inversely correlated with variables with opposite signs. That is, YLD showed direct correlation with the CCI in R6 and R8, Chl total in R8 and DM, and all these variables showed inverse correlation with PCG. With lower intensity, YLD also showed a direct correlation with HGW (−0.28), PCL (−0.44) and NPP (−0.51). In PC2, the relevant variables were NPP (−0.68) and NGP (0.80), that is, NPP was inversely correlated with NGP.

The biplot graph shows that the treatments with the highest doses of P_2_O_5_ (40 and 60 kg ha^−1^) and PSB (100 to 300 mL ha^−1^) were located close to the variables related to the highest agronomic performance, physiological capacity and yield of common beans. On the other hand, the control treatment and the others with the lowest doses of P_2_O_5_ and PSB had higher PCG and were located in opposite quadrants to the variables of agronomic performance, physiological capacity and yield.

## 3. Discussion

### 3.1. Growth and Photosynthetic Efficiency Promoted by PSB and Phosphate Fertilization Doses

PSB and phosphate fertilization improved the physiology and growth of the common bean, which potentially suggests that there was a change in the dynamics of P availability to common bean plants caused by treatments that provide P. PSB inoculation in the absence and at moderate doses of phosphate fertilization increased the content of photosynthetic pigments, promoting the production of chlorophylls. In addition to their effects on P solubilization, bacteria of the genus *Bacillus* also have the ability to fix N [26], a nutrient that is present in the structure of chlorophyll [27]. The production of chlorophylls can be increased by a beneficial interaction between plants and bacteria and, consequently, it may have improved the photosynthetic capacity of plants [28]. Adequate P availability can modulate the activity of RuBisCO, a key protein of photosynthesis, which is also associated with improved absorption of other nutrients, especially magnesium (Mg), which is the central atom of chlorophyll molecules [27,29]. A previous study indicated that higher concentrations of Mg and chlorophyll occurred in a legume crop (*Glycine max* L.) when it was inoculated with *B. subtilis* and *B. megaterium* [26]. In addition, photosynthetic pigments may have been modulated due to the ability of *Bacillus* to alter plant physiology through the synthesis of phytohormones [30]. Indole-acetic acid (IAA) is the most common auxin and is recorded in bacterial activities. In plants, this phytohormone acts on physiological processes by triggering apical dominance, cell division and differentiation, seed germination and root development [31].

For leaf area, the results showed that, in the absence of phosphate fertilization, the common bean plants needed doses greater than 85.66 mL ha^−1^ of PSB to produce maximum leaf area. In this situation, the limitation of P may have stimulated the interaction of the common bean with the bacteria in order to meet the needs of the plant [32]. In addition, linear growth was observed in the leaf area of common beans with phosphate fertilization when plants were inoculated with intermediate doses of PSB. The promotion of leaf expansion may have been influenced by the action of PSB in making P available to plants. Phosphorus has recognized importance in the process of cell division, so adequate availability of this nutrient favors leaf expansion [33]. Inoculation of *Bacillus megaterium* improved the leaf area and total biomass in both leguminous and non-leguminous plants through growth promoting activity [34]. In cases of deficiency, processes such as photosynthesis and respiration are affected, with effects on the quality of the leaves, which become smaller and thinner, and a consequent reduction in plant growth and development [12]. These changes in leaf growth are also attributed to the increase in leaf photosynthetic pigments observed in these treatments (Figure 3).

In general, the maximum integrated application of PSB (300 mL ha^−1^) and P_2_O_5_ (60 kg ha^−1^) treatments caused a decrease in the production of photosynthetic pigments (Figure 3) and a smaller leaf area (Figure 6). This behavior may occur due to microbial immobilization of P at higher rates of inoculation [24]. Microbial biomass activity can adjust to the resources available in the soil, acting as a source in a P-limited environment and as a sink in an environment with higher P concentration [34]. Another plausible event is that the exudation of organic acids, the main mechanism of phosphate solubilization, is stopped by these bacteria in an environment with high P supply [35]. This result suggests that PSB application was more efficient in the absence and under moderate management of phosphate fertilization and less effective with excess P (dose of 60 kg ha^−1^) for the cultivar IAC 2051 in soil with medium P content (32 mg kg^−1^) according Ambrosano et al. [36,37].

The Chlorophyll Content Index (CCI) measured at different phenological stages of the common beans showed linear increases as a function of P_2_O_5_ doses in stages R6 and R8, compared to the CCI measured in V4. As observed, the CCI was not influenced by P_2_O_5_ doses in V4. This could be because at this phenological stage the plants are still small, with low dry matter accumulation. As in this stage the plants do not yet have a high need for nutrients, the P_2_O_5_ doses applied caused no major differences in plant growth. Higher values of this index occurred in the R6 stage, which is expected, since higher plant growth rates occur in this stage. Ref. [38] also observed a higher CCI in the R6 stage in a common bean cultivar of indeterminate growth habit, whose characteristic is the production of vegetative structures until the end of the cycle. In the R8 stage, this index showed a reduction, which occurred due to the beginning of plant senescence and leaf fall.

### 3.2. P Content and Accumulation Promoted by PSB and Phosphate Fertilization Doses

The P content in *Phaseolus vulgaris* plants was influenced by the application of P in the soil (Supplementary Table S1). In the leaves, the P contents obtained are in accordance with the sufficiency range for common beans (2.5 to 4.0 g kg^−1^) [34]. The linear increase in P content observed in the leaves is explained by the higher availability of P provided by increasing doses of phosphate fertilizer [39]. However, there was a reduction in the P content in the dry matter at P_2_O_5_ doses above 5.6 kg ha^−1^, which was also observed in the P content in the grains. This result may be related to the dilution effect of the contents of this nutrient in the total aerial part, due to the increase in plant biomass and grain production at higher doses of phosphate fertilizer (Figure 8A,C) [29]. This can be verified by the multivariate analysis of principal components (Figure 9), in which the PCG was inversely correlated with YLD and DM. Other studies have also reported the effects of nutrient dilution in common bean cultivars that had higher grain production [40].

For the nutrition components of *P. vulgaris* plants, microbial inoculation positively affected the P content and P accumulation in biomass and grains. The strains of *Bacillus megaterium* (CNPMS B119) and *Bacillus subtilis* (CNPMS B2084) were previously isolated and tested for phosphate solubilization efficiency [41,42,43]. In addition to their effect on increasing P availability, *Bacillus* species are multifunctional, with proven activities in the synthesis of growth-promoting compounds (IAA, siderophores, and biofilm) that improve the root system and nutrient exploration [17,44]. Part of the promotion of P accumulation in *Phaseolus vulgaris* biomass and grains may have been improved by the growth-promoting activities of the *Bacillus*. The average reference P content for common beans is 3 kg t^−1^ of grains [37]. The highest P content observed in our study was 4.4 kg t^−1^ of grains when plants were inoculated with 192 mL of PSB, and accumulation and export values are directly related to yield gains [29]. This explains the higher yields achieved in our study when common bean plants were inoculated.

### 3.3. Production Components and Grain Yield Promoted by PSB and Phosphate Fertilization Doses

Inoculation with phosphate-solubilizing bacteria alone had effects on the production components and grain yield of common beans. Parameters such as number of pods, number of grains per pod and grain weight are components of the common bean crop that, in adequate proportion, influence its final grain yield [29]. The NPP component was more sensitive to the inoculation doses. In our study, the absence of inoculation resulted in lower NPP production, but promoted higher NGP; while, under progressive application of PSB doses, the common bean plants produced higher NPP with lower NGP. This inverse relationship is related to the effort made by plants with fewer pods to compensate for production, thus producing more grains per pod [45], which was confirmed by the PC2 of the multivariate principal component analysis (Figure 9). However, higher pod production may be associated with the role played by P in the formation of flowers and pods [29]. Among the production components, number of pods is the one with the highest correlation with yield gains and may be influenced by the genotype factor and management conditions [46].

Dry matter responded to the simple effects of PSB doses and P_2_O_5_ doses, without interaction. Maximum dry matter production was reached at P_2_O_5_ and PSB doses of 49.4 kg ha^−1^ and 186.1 mL ha^−1^, respectively. This result is related to the maximum potential of the plant for converting energy into biomass [21], and directly leads to yield gains [29].

The P provided by the phosphate fertilizer influenced grain yield. Although the production components were not affected by this factor, other differences obtained for this treatment may have been influenced, such as the high nutritional status of the common bean plants (Figure 1), linear increase in the chlorophyll content index (Figure 5) and performance of photosynthetic pigments in the reproductive stages (Figure 4), as well as the interaction between all these components. Multivariate analysis showed that YLD was directly correlated with the CCI in R6 and R8 and with Chl total in R8, corroborating what was previously discussed. Reference [38] found that, in the reproductive stages, the CCI assessment is the one that most correlates with the YLD of crops, as observed in the present study. According to the authors, evaluations in more advanced stages of the cycle allow significant differences in the index according to the application of the treatments, since in this stage plants under less favorable conditions for growth will show lower nutritional and physiological status compared to those under more favorable conditions.

In summary, the gains in dry matter and yield promoted by the use of PSB are similar to those promoted by the use of phosphate fertilizer, associated with the P supply by either chemical or biological fertilization. The gains promoted by the treatments are due to the function performed by P in plants, especially legumes, stimulating nodulation and biological N fixation [47]. As these are important parameters for the final yield, the common bean responded with maximum yield of 3489 kg ha^−1^ when inoculated with a PSB dose of 159 mL ha^−1^ (Figure 8D). Compared to the control (0 mL ha^−1^ of PSB), there was an increase of 389 kg ha^−1^ in YLD, equivalent to almost seven bags of 60 kg of common beans. The maximum grain yield (3498 kg ha^−1^) achieved in this study with PSB is above the national average yield (1532 kg ha^−1^) and the average of the São Paulo state in the autumn-winter season (2474 kg ha^−1^) [48]. These results are important and provide a new approach to sustainable common bean production.

### 3.4. Common Bean Grain Quality Promoted by Phosphate Fertilization Doses

As the common bean is a food crop, management should be focused on the final product that will be made available to the market [40]. In summary, the treatments did not influence grain quality, except for cooking time. The averages obtained for each attribute are within the average range for the cultivar, as required by the consumer market. The average yield of sieves ≥12 in the experiment was 85.7%, indicating the formation of large grains above the reference average required by common bean packing companies, which is 70% [49].

For cooking time, the reduced dose of phosphate fertilization (29.7 kg ha^−1^ of P_2_O_5_) was determinant for reducing the resistance to cooking. It was also observed that doses higher than that led to longer cooking times. For the P content in the grains, it was observed that they were lower under excessive doses of phosphate fertilization (Figure 1C). A different result was obtained by [44], who found that genotypes with higher concentrations of P had longer cooking time.

Grain quality has high variability and is directly related to genetic factors, crop management conditions and their interactions. Agricultural practices such as crop succession [50] and growing season [51] can alter the quality of common bean grains. In general, the grains showed high susceptibility to cooking (<16 min) according to the cooking resistance scale of [52]. Bean grains that can be cooked in 15 to 30 min are preferred by the consumer market, due to lower energy expenditure in their preparation [53].

## 4. Materials and Methods

### 4.1. Experimental Area

The experiment was conducted in the winter season (3rd crop season) of the 2021/2022 agricultural year, at the São Paulo State University (Unesp), Jaboticabal—SP, Brazil, in an area located near the coordinates 21°14′59″ S, 48°17′16″ W and at an altitude of 570 m (Supplementary Figure S1). The region has an Aw climate, according to Köppen’s climate classification. This climate type (Aw) is characterized by hot, rainy summers and hot, dry winters. The average annual rainfall in the region is 1425 mm, with an average temperature of 22 °C. Historically, the experimental area has been used for the cultivation of annual crops (cereals and legumes).

The soil is classified as Oxisol [54], clayey in texture, with a gently undulating relief and a 6% slope. Before the experiment, soil samples were collected to determine fertility in the first 20 cm of the surface layer (Table 1), according to Raij et al. [55]. Climatological data were recorded throughout the experimental period (Supplementary Figure S2). The mean maximum and minimum temperatures during the experimental period were 27.9 and 13.4 °C, respectively, with accumulated precipitation of 45.6 mm.

### 4.2. Experimental Design and Treatments

The experimental design chosen was randomized blocks (RBD), in a 4 × 4 factorial scheme, with four replicates. The experimental factors consisted of four doses of P_2_O_5_, in addition to the recommended dose; there were also three other types of management: one with half of the recommended dose, one with a 50% increase and a control without fertilization (0, 20, 40 and 60 kg ha^−1^) and four doses of phosphate-solubilizing bacteria (PSB)-based inoculant (0, 100, 200 and 300 mL ha^−1^).

According to the soil analysis (Table 1) and considering expected yields higher than 2500 kg ha^−1^, the recommendation was to apply 40 kg ha^−1^ of P_2_O_5_ [34]. The source of P_2_O_5_ used was triple superphosphate (Fertipar^®^), which contained 41% of P_2_O_5_. The inoculant was the commercial product BiomaPhos^®^, which contains the PSB strains *Bacillus subtilis* (CNPMS B2084—BRM034840) and *Bacillus megaterium* (CNPMS B119—BRM033112), with a concentration of 4 × 10^9^ viable cells mL^−1^.

### 4.3. Crop Practices

The experiment was conducted using the newly released common bean cultivar IAC 2051, which has the following characteristics: indeterminate growth habit, type II, normal cycle, average grain cooking time of 30 min and average protein content of 20% [56]. Plant population was adjusted to 240,000 plants ha^−1^. Previously, all common bean seeds were inoculated with *Rhizobium tropici*, using a liquid commercial inoculant (BiomaRhyzo^®^ “Fazenda Rio Grande, PR, Brazil”), SEMIA 4077 strain, with 4 × 10^9^ viable cells mL^−1^, at a dose of 150 mL for each 50 kg of seeds. After drying the seeds, inoculation for phosphate solubilization was performed according to the treatments. Both inoculations were performed by coating and mixing the inoculants and common bean seeds in plastic bags, manually.

For setting up the experiment, the sowing furrows were mechanically opened. Each experimental unit had 5 rows of common bean plants, each 5 m in length, spaced 0.45 m apart. Subsequently, sowing fertilization was performed manually, using 100 kg ha^−1^ of the 20-00-20 formulation, providing 20 kg ha^−1^ of N and 20 kg ha^−1^ of K_2_O, along with the respective P_2_O_5_ doses of each treatment. Manual sowing was carried out in the furrows.

In the top-dressing fertilization, the N rate was of 90 kg ha^−1^ splitting two times. In the first application (17 days after emergence—DAE), ammonium sulfate fertilizer was used, aiming to supply S to the common bean plants in addition to N. In this first application, 30 kg ha^−1^ of S and 28 kg ha^−1^ of N were provided [37]. In the second application (28 DAE), urea was used as a source of N, with a dose of 60 kg ha^−1^ of N. The applications were performed in a continuous strip 10 cm away from the center of the common bean rows. Irrigation was applied by conventional sprinklers, with variable irrigation intervals according to the need of the crop. The total volume applied was 440 mm.

### 4.4. Traits Evaluated

During the experiment, physiological (chlorophyll content index (CCI), chlorophyll a (Chl a), chlorophyll b (Chl b), total chlorophyll (Chl total) and carotenoids (Cx + c)), nutritional (phosphorus content in leaves; phosphorus content in dry matter (R6); phos-phorus content in grains, phosphorus accumulation in dry matter (R6); P export at physio-logical maturity (R9) and agronomic efficiency) and production attributes (leaf area, dry mass, number of pods per plant, number of grains per pod, hundred-grain weight, grain yield) were evaluated in the plants and qualitative attributes were evaluated in the grains. At 37, 54 and 79 DAE, referring to the phenological stages V4 (third fully expanded trifoliate leaf), R6 (full flowering) and R8 (grain filling), the concentrations of chlorophyll a (Chl a), chlorophyll b (Chl b), total chlorophyll (Chl total) and carotenoids (Cx + c) were determined [57]. Subsequently, absorbance readings were recorded at 470 nm, 645 nm and 663 nm using a spectrophotometer (Beckman Coulter DU 640, Brea, CA, USA) to calculate pigment content in mg g^−1^ of leaf fresh matter using the equations suggested by Lichtenthaler [57]. At 28, 49 and 77 DAE, referring to stages V4, R6 and R8, respectively, readings of the chlorophyll content index (CCI) were performed using the portable chlorophyll meter CCM-200 Plus (Opti-Sciences Inc., Hudson, NH, USA). Readings were performed considering the third fully expanded trifoliate leaf from the apex, in 9 trifoliate leaves per plot.

To determine P content in the leaves (PCL), 5 trifoliate leaves with petiole at stage R6 were collected in each plot, in the middle third of five plants, according to the methodology described by [37]. The leaves were washed (using water with 1% detergent and deionized water) and then dried in a forced air circulation oven at a temperature of 65 to 70 °C until reaching constant mass. After drying, they were ground, and P content was determined using the method described by Malavolta et al. [58]. At full flowering (R6), leaf samples were collected from three plants of the usable plot for subsequent calculation of the leaf area index (LAI) using the area meter LI-COR 3100C (Biosciences, Lincoln, NE, USA).

To determine the dry matter (DM) of the aerial parts of the common beans, five consecutive plants were collected during the phenological stage R6 in the usable area of each plot. Then, the plants were washed with distilled water and dried in a forced circulation oven at 65 °C until reaching constant mass for subsequent weighing. The samples used for DM determination were ground in a Wiley-type mill and then the P content [53] and P accumulation in the aerial part were determined (PCDM and PADM, respectively).

At the physiological maturity stage (R9), ten plants were collected from the usable area of each plot to determine the following production components: number of pods per plant (NPP), number of grains per pod (NGP) and hundred-grain weight (HGW). HGW was determined by weighing four subsamples of 100 grains from each experimental plot, and correcting the data to moisture content of 0.13 kg kg^−1^ on a wet basis.

Harvest was carried out by manually uprooting the plants present in three rows of the usable area of each plot. After drying in the sun, mechanized threshing was performed. To obtain the yield (YLD), grain moisture was standardized to 0.13 kg kg^−1^ on a wet basis. After harvest, P content in the grains (PCG) was determined according to [58], as was P export. P export was calculated based on the data for yield and P content in the grains. The agronomic efficiency (AE) of the treatments was obtained by the following equation: AE = (YLD_WP_ − YLD_WoP_)/(QP), where YLD_WP_ = grain yield with phosphate fertilizer; YLD_WoP_ = grain yield without phosphate fertilizer; and QP = quantity of P_2_O_5_ applied [29].

After collection, the grain samples were placed in paper bags and stored for 30 days at ambient temperature to determine the qualitative attributes. Initially, the grains were classified according to size by passing the sample from each plot through a set of sieves with oblong holes: 11/64″ × 3/4 (4.37 × 19.05 mm), 12/64″ × 3/4 (4.76 × 19.05 mm), 13/64″ × 3/4 (5.16 × 19.05 mm), 14/64″ × 3/4 (5.56 × 19.05 mm) and 15/64″ × 3/4 (5.96 × 19.05 mm). The ratio of the mass of grains retained on each sieve to the total mass was used to calculate the yield of sieves greater than or equal to 12 (YS ≥ 12). Grains retained on sieve 13 were used to determine the other qualitative attributes of the grains (crude protein content (CPC), cooking time (CKT), time for maximum hydration (TMH) and hydration ratio (HR)).

The time for maximum grain hydration (TMH) was determined using samples of 50 g of grains from each plot, with an initial volume of 200 mL of distilled water. Every two hours, until 18 h, the volume of water not absorbed by the grains was quantified. At the end of the expected time for hydration, the samples were weighed again. TMH was determined by polynomial regression between time (hours) and hydration capacity (mL). Hydration ratio (HR) was determined by the ratio between the final mass and the initial mass of grains in the samples used in TMH evaluation. The crude protein content (CPC) of the grains was determined by the equation: CPC = 6.25 × N, where N is the nitrogen content in the grains determined by sulfuric digestion and semi-micro-Kjeldahl method [58].

To determine the cooking time (CKT), grain samples were previously hydrated for 12 h. Subsequently, the grains were arranged in a Mattson cooker. This cooker consists of 25 vertical plungers, each of which under a 90 g weight and with a tip of 1 mm in diameter. This structure rests on the grains during cooking, and when each grain is cooked, the tip penetrates it. The final time taken to cook the sample was computed as the moment when 50% + 1, that is 13, plungers were displaced. During the test, water temperature was maintained at 96 °C. The scale of Proctor and Watts [52] was adopted to assess the level of resistance to cooking.

### 4.5. Statistical Analysis

Due to the dependence of the original data set, the data were subjected to multivariate exploratory analysis by principal components [59]. For this, a correlation matrix was initially created to select the variables with greater dependence on the others within the data set. The variables used in this analysis were YLD, DM, HGW, NPP, NGP, PCG, PCL and the CCI in R6 and R8, and Chl total in R8. Prior to the analysis, the data were standardized (zero mean and unit variance). The two-dimensional scatter plot (biplot) was created using the first two principal components that showed eigenvalues above 1.00 [60]. The covariance matrix of the original variables was used to extract the eigenvalues. Variables with scores (factor loadings) above 0.60 were considered relevant within each principal component. In the text, the values within parentheses represent the value of the factor loading with the respective principal component. Multivariate analysis was performed using Statistica^®^ software v. 7.0 (StatSoft, Tulsa, OK, USA).

In the univariate analysis, the obtained data were subjected to ANOVA assumption tests (normality and homogeneity) and then to analysis of variance by the F test (*p* < 0.05). Since the study factors were quantitative, when the data were significant in the ANOVA, regression models were fitted using R software v.4.3.1 (R Development Core Team, 2015).

## 5. Conclusions

Although there was no interaction between P_2_O_5_ and PSB doses, these two studied factors increased production components and yield of common beans, P content in the leaves, P accumulation, number of pods per plant, dry matter and yield. P_2_O_5_ application increased yield by 79 kg ha^−1^ for each 10 kg ha^−1^ of P_2_O_5_ added, while inoculation with PSB (dose of 159 mL ha^−1^) increased yield by 12% (389 kg ha^−1^) compared to the management without inoculation. The improvement of the growth and physiological and nutritional status of the common bean due to P_2_O_5_ application and PSB inoculation was fundamental to the increase in yield, so these are alternatives for common bean production.

## Figures and Tables

**Table 1:** Soil chemical characterization in the 0.00–0.20 m layer.

Layer	P	S		SOM		pH	K	Ca	Mg	H + Al	Al	SB
(m)	mg kg^−1^			g kg^−1^		-	mmol_c_ kg^−1^					
0.00–0.20	32.0	5.0		26.0		5.8	6.4	24.0	14.0	18.0	0	44.4
	B		Cu	Fe	Mn	Zn	CEC			V		m
	mg kg^−1^						mmol_c_ kg^−1^			%		
	0.86		11.8	24.0	2.7	4.5	62.4			71.0		0

P, K, Ca and Mg: determined by resin method; SOM: soil organic matter, content determined by potassium dichromate oxidation method; pH: determined in CaCl_2_ solution; H  +  Al: potential acidity determined by Shoemaker-McLean-Pratt (SMP) buffer method; SB: sum of bases; B determined by hot water method; Cu, Fe, Mn and Zn determined by diethylene triamine penta-acetic acid—DTPA; CEC: cation exchange capacity; V: base saturation; m: Al saturation.

## Data Availability

Data are contained within the article or Supplementary Materials.

## Supplementary Materials

The following supporting information can be downloaded at: https://www.mdpi.com/article/10.3390/plants12223827/s1, Figure S1: Description of the study area. Map of South America highlighting Brazil (A); State of São Paulo (B); city of Jaboticabal (C) and experimental area (D); Figure S2: Average rainfall, maximum, average and minimum temperatures recorded during the period in which the experiment was conducted (20 April to 27 August 2022). Jaboticabal, Sao Paulo, Brazil. Sowing (S), Emergence (V1), Third fully expanded trifoliate leaf (V4), Full flowering (R6) and Physiological maturity (R9); Figure S3: Common bean cooking time (CKT) as a function of P2O5 doses; Table S1: F values for phosphorus content and accumulation in common bean as a function of P2O5 and phosphate solubilizing-bacteria (PSB) doses; Table S2: F values for chlorophyll content index (CCI), chlorophyll a (Chl a), chlorophyll b (Chl b), total chlorophyll (Chl total) and carotenoids (Cx + c) as a function of P2O5 and phosphate solubilizing-bacteria (PSB) doses in the stages V4, R6 and R8; Table S3: F values for leaf area (LA), dry mass (DM), number of pods per plant (NPP), number of grains per pod (NGP), hundred-grain weight (HGW), grain yield (YLD) as a function of P2O5 and phosphate solubilizing-bacteria (PSB) doses; Table S4: F Values for yield of sieves ≥ 12 (YS ≥ 12), crude protein content (CPC), cooking time (CKT), time for maximum hydration (TMH) and hydration ratio (HR) of common bean grains as function of P2O5 and phosphate solubilizing-bacteria (PSB) doses.
