# Supplementary material for: Application of Bacillus spp. Phosphate-Solubilizing Bacteria Improves Common Bean Production Compared to Conventional Fertilization
Source: Plants (Basel). 2023 Nov 11;12(22):3827. doi: 10.3390/plants12223827 (PMC10675661; doi:10.3390/plants12223827)
Supplement: Supplementary file 1 [file plants-12-03827-s001.zip › plants-2619360-supplementary.pdf]

plants

*Supplementary Materials*

**Table S1.** F values for phosphorus content and accumulation in common bean as a function of $P_2O_5$ and phosphate solubilizing-bacteria (PSB) doses.

| F value | PCL | PCDM | PCG | PADM | Export | AE |
|---|---|---|---|---|---|---|
| | | g kg$^{-1}$ | | | kg ha$^{-1}$ | kg kg$^{-1}$ |
| $P_2O_5$ doses (P) | 3.52* | 5.71** | 3.75 * | 1.68$^{ns}$ | 0.98 $^{ns}$ | 1.21 $^{ns}$ |
| PSB doses (I) | 0.27$^{ns}$ | 2.93* | 0.26 $^{ns}$ | 5.27 ** | 3.95 * | 2.53 $^{ns}$ |
| P x I | 1.00$^{ns}$ | 1.68$^{ns}$ | 0.91 $^{ns}$ | 1.87 $^{ns}$ | 0.56 $^{ns}$ | 1.90 $^{ns}$ |
| Mean | 2.9 | 2.9 | 4.26 | 4.58 | 14.3 | 9.79 |
| CV (%) | 10.29 | 7.54 | 6.65 | 19.05 | 13.03 | 74.59 |

**, * and ns: significant at $p < 0.01$, $p < 0.05$ and not significant, respectively. CV: coefficient of variation; PCL: phosphorus content in leaves; PCDM: phosphorus content in dry matter ($R_6$); PCG: phosphorus content in grains: PADM: phosphorus accumulation in dry matter ($R_6$); P export at physiological maturity ($R_9$) and AE: agronomic efficiency.

**Table S2.** F values for chlorophyll content index (CCI), chlorophyll a (Chl a), chlorophyll b (Chl b), total chlorophyll (Chl total) and carotenoids (Cx + c) as a function of $P_2O_5$ and phosphate solubilizing-bacteria (PSB) doses in the stages V4, R6 and R8.

| F value | Vegetative stage (V4) | | | | |
|---|---|---|---|---|---|
| | CCI | Chl a | Chl b | Chl total | Cx + c |
| | ---------------------------mg g$^{-1}$ -------------------------- | | | | |
| $P_2O_5$ doses (P) | 1.44 $^{ns}$ | 3.66 ** | 10.56 ** | 3.80 * | 2.95 ** |
| PSB doses (I) | 0.3 $^{ns}$ | 4.48 * | 3.50 * | 0.39 $^{ns}$ | 0.41 $^{ns}$ |
| P x I | 1 $^{ns}$ | 8.32 ** | 6.62 ** | 1.5 $^{ns}$ | 2.28 * |
| Mean | 19.06 | 0.21 | 0.3 | 0.52 | 0.46 |
| CV (%) | 7.76 | 8.07 | 7.19 | 3.92 | 14.5 |
| F value | Reproductive stage (R6) | | | | |
| | CCI | Chl a | Chl b | Chl total | Cx + c |
| $P_2O_5$ doses (P) | 7.46 ** | 0.28 $^{ns}$ | 0.40 $^{ns}$ | 0.35 $^{ns}$ | 0.61 $^{ns}$ |
| PSB doses (I) | 0.64 $^{ns}$ | 0.75 $^{ns}$ | 1.40 $^{ns}$ | 1.12 $^{ns}$ | 1.56 $^{ns}$ |
| P x I | 0.63 $^{ns}$ | 0.83 $^{ns}$ | 0.90 $^{ns}$ | 0.86 $^{ns}$ | 1.42 $^{ns}$ |
| Mean | 22.18 | 0.22 | 0.33 | 0.54 | 0,5 |
| CV (%) | 7.28 | 11.32 | 11.36 | 11.28 | 11.24 |
| F value | Reproductive stage (R8) | | | | |
| | CCI | Chl a | Chl b | Chl total | Cx + c |
| $P_2O_5$ doses (P) | 3.79 * | 4.21 * | 7.03 ** | 3.70 * | 2.22 $^{ns}$ |
| PSB doses (I) | 0.12 $^{ns}$ | 0.52 $^{ns}$ | 0.78 $^{ns}$ | 0.70 $^{ns}$ | 0.80 $^{ns}$ |
| P x I | 0.82 $^{ns}$ | 0.96 $^{ns}$ | 1.32 $^{ns}$ | 0.86 $^{ns}$ | 0.80 $^{ns}$ |
| Mean | 21.6 | 0.15 | 0.26 | 0.41 | 0.47 |
| CV (%) | 10.79 | 16.35 | 9.77 | 11.35 | 13.93 |

**, * and ns: significant at $p < 0.01$, $p < 0.05$ and not significant, respectively. CV: coefficient of variation.

**Table S3.** F values for leaf area (LA), dry mass (DM), number of pods per plant (NPP), number of grains per pod (NGP), hundred-grain weight (HGW), grain yield (YLD) as a function of $P_2O_5$ and phosphate solubilizing-bacteria (PSB) doses.

| **F value** | **LA** | **DM** | **NPP** | **NGP** | **HGW** | **YLD** |
|---|---|---|---|---|---|---|
| | **cm²** | **g planta⁻¹** | **-----------nº---------** | | **g** | **kg ha⁻¹** |
| $P_2O_5$ doses (P) | 0.55 $^{ns}$ | 6.65 ** | 1.49 $^{ns}$ | 1.49 $^{ns}$ | 0.50 $^{ns}$ | 5.54 ** |
| PSB doses (I) | 3.63 * | 3.57 * | 7.83 ** | 3.78 * | 0.19 $^{ns}$ | 5.13 ** |
| P x I | 2.73 * | 0.90 $^{ns}$ | 1.92 $^{ns}$ | 1.01 $^{ns}$ | 1.23 $^{ns}$ | 0.18 $^{ns}$ |
| Mean | 1065.18 | 7.29 | 12.22 | 4.91 | 26.27 | 3295.38 |
| CV (%) | 19.11 | 15.44 | 15.96 | 14.43 | 2.25 | 10.67 |

**, * and ns: significant at $p < 0.01$, $p < 0.05$ and not significant, respectively. CV: coefficient of variation.

**Table S4.** F Values for yield of sieves ≥ 12 (YS ≥ 12), crude protein content (CPC), cooking time (CKT), time for maximum hydration (TMH) and hydration ratio (HR) of common bean grains as function of $P_2O_5$ and phosphate solubilizing-bacteria (PSB) doses.

| F value | YS ≥ 12 | CPC | CKT | TMH | HR |
|---|---|---|---|---|---|
| | ------------------ % --------------- | | min:seg | h:min | - |
| $P_2O_5$ doses (P) | 0.92 $^{ns}$ | 2.01 $^{ns}$ | 4.20 * | 0.05 $^{ns}$ | 0.14 $^{ns}$ |
| PSB doses (I) | 1.36 $^{ns}$ | 1.26 $^{ns}$ | 0.67 $^{ns}$ | 1.30 $^{ns}$ | 0.69 $^{ns}$ |
| P x I | 1.02 $^{ns}$ | 0.87 $^{ns}$ | 0.78 $^{ns}$ | 1.25 $^{ns}$ | 0.93 $^{ns}$ |
| Mean | 85.7 | 17.0 | 12:23 | 15:25 | 2.02 |
| CV (%) | 3.14 | 5.56 | 7.67 | 15.05 | 1.84 |

* and ns: significant at $p < 0.05$ and not significant, respectively.

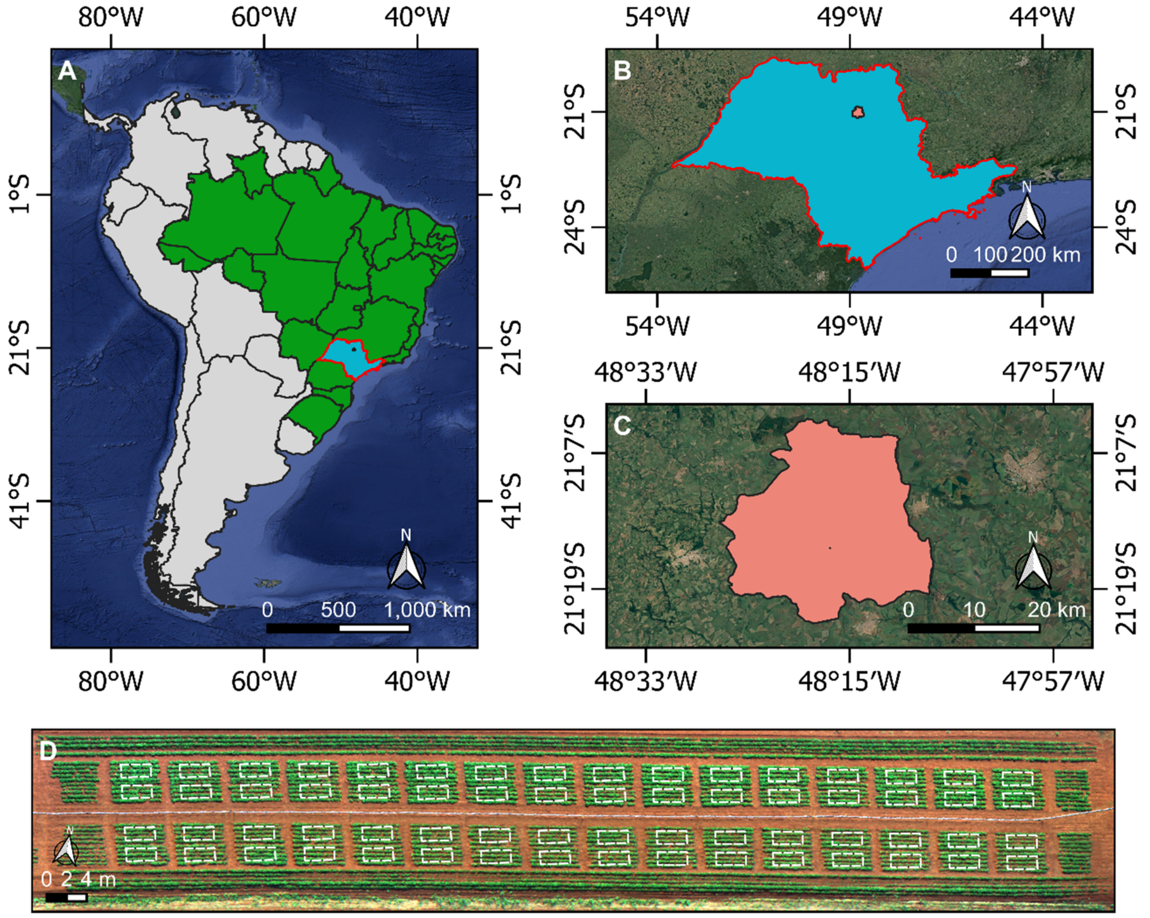


**Figure S1.** Description of the study area. Map of South America highlighting Brazil (A); State of São Paulo (B); city of Jaboticabal (C) and experimental area (D).

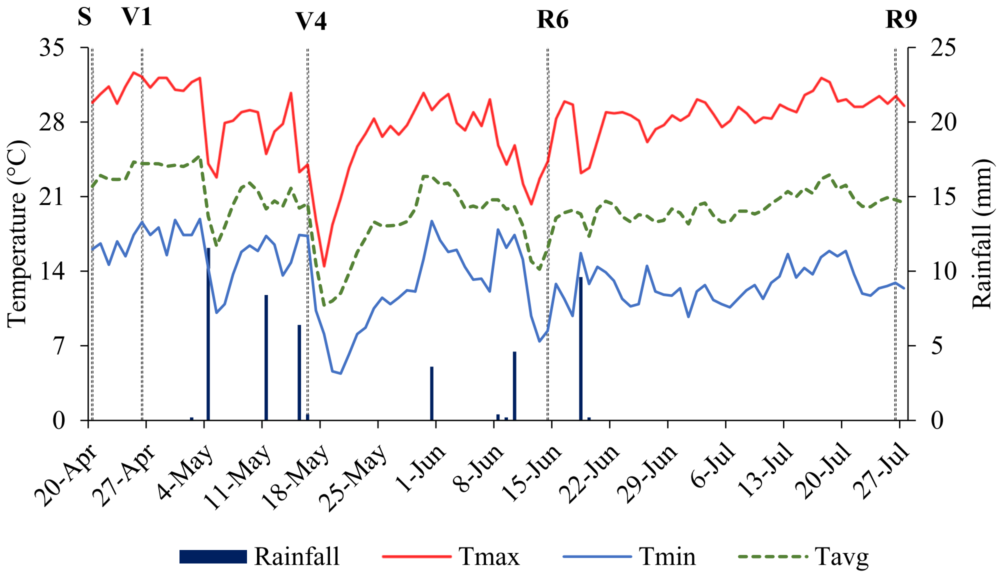


**Figure S2.** Average rainfall, maximum, average and minimum temperatures recorded during the period in which the experiment was conducted (20 Apr. to 27 Aug. 2022). Jaboticabal, Sao Paulo,

Brazil. Sowing (S), Emergence (V1), Third fully expanded trifoliate leaf (V4), Full flowering (R6) and Physiological maturity (R9).

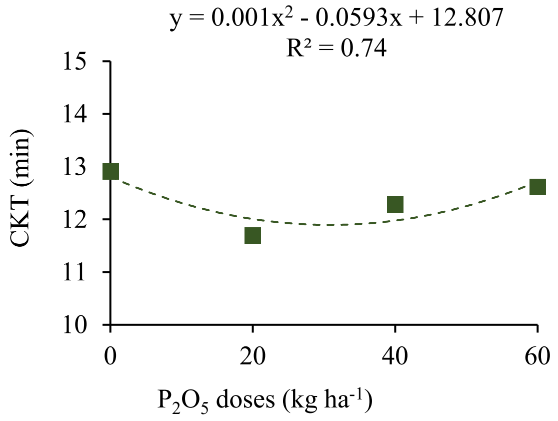


**Figure S3.** Common bean cooking time (CKT) as a function of $P_2O_5$ doses.
